# Attitudes, Perceptions, and Knowledge of Greek Midwives Toward LGBT Individuals: Findings From the ATHENA Study and Validation of the Greek Version of the Perception and Knowledge of Sexual and Gender Minority Health (PKSGMH) Questionnaire

**DOI:** 10.7759/cureus.86632

**Published:** 2025-06-23

**Authors:** Angeliki Antonakou, Vikentia C Harizopoulou, Eleni Theodoridou, Kalliopi Gkougkousidou

**Affiliations:** 1 Department of Midwifery, School of Health Sciences, International Hellenic University, Thessaloniki, GRC; 2 Department of Midwifery, Faculty of Health and Caring Sciences, University of West Attica, Athens, GRC

**Keywords:** attitudes, education, gender identity, greece, knowledge, lgbt, midwifery, sexual and gender minorities, sexual orientation

## Abstract

Introduction: Lesbian, gay, bisexual, and transgender (LGBT) people experience worse health indicators compared to the general population and encounter notable healthcare disparities, especially in reproductive and midwifery care. However, the growing visibility of the LGBT community and the ongoing advocacy for reproductive equality have raised awareness about nontraditional families and led to an increased demand for midwifery care that is inclusive and responsive to these needs. This study aimed to investigate Greek midwives' knowledge and attitudes toward LGBT health by validating the Greek version of the Perception and Knowledge of Sexual and Gender Minority Health (PKSGMH) instrument.

Methods: An anonymous online survey on n = 187 Greek midwives was conducted from July to August 2024. The PKSGMH questionnaire was used after obtaining the author's permission and adapting it to align with Greek standards. It consisted of 81 questions, including eight demographic questions, 66 questions related to LGBT issues, and seven questions about professional midwifery experience.

Results: Participants’ mean age was 41 years (standard deviation (SD) = 9.9). One hundred eighty-four were women (99.5%), n = 181 (96.8%) were heterosexual, n = 125 (66.8%) were married, and n = 131 (70.1%) had LGBT acquaintances. One hundred twelve (59.9%) provided care to LGBT patients, primarily lesbians (52.9%). The participants had an average professional experience of 15.2 years (SD = 9.9), but only eight (4.3%) had received training specifically for LGBT individuals. However, n = 140 (76.1%) came into contact with gay, lesbian, and bisexual patients. Reliability indexes of all scales were above or close to acceptable levels (≥0.70). Positive correlations were found among attitude scales toward LGBT people. Negative attitudes toward LGBT people were linked to more negative attitudes toward LGBT patients and less practice with them. Greater knowledge was associated with more positive attitudes and better practice with LGBT patients. Being married or partnered in a heterosexual relationship, not having LGBT acquaintances, and a lack of experience in providing care to LGBT patients were associated with having more negative attitudes toward LGBT people. Midwives with LGBT-specific training had less negative attitudes. Older age and longer years of professional experience were associated with more negative attitudes. At the same time, more frequent contact with LGBT people/patients in the workplace was associated with less negative attitudes.

Conclusion: The Greek version of the PKSGMH instrument demonstrated good reliability and validity. Greek midwives have moderate to high levels of knowledge and neutral to positive attitudes toward LGBT people and patients. Continuing professional education on LGBT-focused issues could resolve knowledge gaps, misconceptions, and perceived competency deficiencies.

## Introduction

In recent decades, sexual and gender minority health has emerged as a critical area of public health research worldwide [1]. This focus has brought issues of people across the spectrum of diverse genders, sexualities, and sex characteristics (LGBTQIA+) to the forefront of public discourse, particularly regarding discrimination, marginalization, and social stigma [1]. The nomenclature’s expansion led to the LGBTQIA+ acronym encompassing lesbian, gay, bisexual, transgender, queer/questioning, intersex, asexual, and other sexual orientations and gender identities, represented by the “+” to include additional identities beyond these terms. The acronym LGBT will be used throughout the article when referring to the above population since there is no universally agreed-upon abbreviation [2].

It is estimated that 9-10% of the global population, approximately 725 million individuals, identify as LGBT [3]. In Greece, the LGBT population is estimated to range between 500,000 and 800,000 people. The prevalence of self-identified LGBT individuals varies notably across countries and generations. For example, recent global surveys indicate that among Generation Z (born after 1997), 18% identify as LGBT, compared to only 7% among Generation X (born between 1965 and 1980) [3]. This generational shift suggests a growing proportion of LGBT individuals within the reproductive-age population, reinforcing the urgent need to ensure inclusive sexual and reproductive health services, including midwifery care. Furthermore, a growing body of research highlights that a significant number of individuals who identify as sexually or gender diverse express a desire to become parents [4,5]. This trend has critical implications for perinatal care, fertility counselling, and reproductive health services.

Multiple systematic reviews and meta-analyses have documented substantial disparities in both mental and physical health among LGBT populations. For example, Meyer’s minority stress model meta-analysis detailed elevated rates of depression and anxiety [6], while Haarmann et al. [7] identified increased prevalence of chronic physical conditions, such as cardiovascular disease and diabetes, in sexual minority women compared to heterosexual peers. This recent meta-analysis on nonbinary youth also highlighted significantly poorer mental health outcomes compared to cisgender counterparts, including higher rates of suicidal ideation and self-harm [7]. Together, these findings underscore the urgent need for culturally competent, inclusive healthcare delivery for LGBT individuals. Yet, despite growing international attention, research on the preparedness of maternal and newborn healthcare providers, particularly midwives, to meet the needs of LGBT individuals remains scarce. The health issues faced by LGBT individuals are closely tied to their sexual and reproductive health care, as both involve the autonomy of individuals in making personal decisions [8]. At the same time, research suggests that lesbian and bisexual women experience barriers to accessing and/or receiving suboptimal fertility and reproductive and maternity healthcare [4,9] and display worse pregnancy success rates and perinatal outcomes, such as preterm birth, miscarriage, and stillbirth, compared to heterosexual women [7,10]. Despite the increasing recognition of the importance of healthcare inclusivity, research within midwifery remains limited, often focusing narrowly on specific aspects of the issue. Internationally, mixed-methods research from the United Kingdom and Ireland reported that many midwives lack confidence in discussing sexual and gender diversity, despite expressing generally positive attitudes toward LGBT clients [11]. In Canada, qualitative interviews with practicing midwives underscored the pivotal role of midwifery advocacy in creating affirming birth spaces for queer and trans parents, while also revealing gaps in communication training [12].

In contrast, Greek research on midwives’ roles in LGBT care is minimal. A recent article in the European Journal of Midwifery explored whether midwifery education in Greece could improve knowledge and attitudes toward LGBTQ people, suggesting a general lack of preparedness among practitioners and identifying education as a key factor in improving care standards [8]. This limited body of evidence highlights the urgent need for formal training and validated assessment tools to support inclusive midwifery practice in Greece for individuals within the LGBT community.

Theoretical framework and research gap

Grounded in Cultural Competency Theory [13] and Implicit Bias Theory [14], this study examines how midwives’ attitudes, knowledge, and the broader systemic context influence the quality of care provided to sexual and gender minority clients. While the Perception and Knowledge of Sexual and Gender Minority Health (PKSGMH) instrument [15] has been validated in English‑speaking contexts [16], it has not yet been adapted or tested in resource-constrained or culturally distinct environments such as Greece. To our knowledge, no study has explicitly assessed Greek midwives’ competence regarding LGBT healthcare, leaving a crucial gap in understanding how professional education and attitudes translate into practice within this predominantly Orthodox sociocultural environment. At the international level, Greece is ranked 35th globally in terms of LGBT rights, reflecting its progress in integrating legal and institutional reforms that support the LGBT community. Notably, in 2024, Greece became the first predominantly Orthodox country to legalize same-sex marriage, and its Parliament extended equal parental rights to same-sex couples, including the right to adopt children [17]. However, despite these legislative advancements, public attitudes have not evolved at the same pace. Recent data suggest that societal acceptance of LGBT individuals in Greece remains limited, revealing a disconnect between policy reform and social perception [18]. This explorative pilot study aimed to investigate Greek midwives’ knowledge and attitudes toward LGBT individuals and their health by translating and validating the Greek version of the PKSGMH instrument and assessing its reliability for use by midwives.

## Materials and methods

Study objectives

Our study, as part of "A meTHod to improvE the knowledge aNd Attitudes" (ATHENA) initiative, supported by the Hellenic Foundation for Research and Innovation (HFRI) under the "Basic Research Financing" call, part of Greece's National Recovery and Resilience Plan "Greece 2.0," funded by the European Union NextGenerationEU, aims to (1) translate and culturally adapt the PKSGMH into Greek following Beaton’s cross‑cultural adaptation guidelines [19]; (2) evaluate its psychometric properties, constructing validity, internal consistency, and test‑retest reliability-among Greek licensed midwives; and (3) provide an initial overview of Greek midwives’ knowledge and attitudes regarding LGBT care. By elucidating midwives’ preparedness to provide inclusive, nonjudgmental care, this study seeks to inform future curricula and policy initiatives aimed at reducing health disparities in sexual and gender minority populations.

Method

A cross-sectional, observational design was selected as the most appropriate method for the exploratory and instrument validation aims of this study. Our primary objective was to assess the psychometric properties (reliability and validity) of the Greek-translated PKSGMH instrument and to provide an initial overview of Greek midwives’ knowledge and attitudes regarding LGBT care. An anonymous online survey was conducted from July to August 2024, published on social media, the official ATHENA study website, and the websites of the nine Greek midwifery professional bodies. All licensed midwives practicing in Greece were eligible to participate. The only requirements were proficiency in Greek and willingness to participate in the study. A priori power analysis was conducted to determine the required sample size. It was calculated that a sample of 180 participants or more would provide the study with 95% power to perform a linear regression model at a significance level of 0.05 and for effect sizes of 0.15 or greater.

Ethical consideration

The study complied with all relevant national regulations and institutional policies, adhering to the principles outlined in the Declaration of Helsinki. Moreover, the study protocol was approved by the International Hellenic University’s Research Ethics Committee (protocol number 31/2024-09/04/2024).

On the first page of the online questionnaire, all participants were informed in detail about the survey’s objective. A “click-if-you-agree” button obtained informed consent. No personal identification information was collected. All data were collected, stored, and analyzed in accordance with applicable data protection regulations, with strict measures taken to ensure the anonymity and confidentiality of all participants throughout the research process.

Translation and cultural adaptation

Permission to translate and validate the PKSGMH instrument for use in the Athena study was formally granted by the original instrument’s creator, Row, via email correspondence [14]. The PKSGMH was translated and culturally adapted into Greek, following internationally recognized guidelines for the cross-cultural adaptation of self-report measures [19], which included the following steps: (a) Two native Greek speakers fluent in English independently translated the original 74-item PKSGMH instrument. (b) Four bilingual midwives reconciled the two translations into a single Greek version through discussion and consensus. Although formal interrater agreement indices were not calculated, discrepancies between the two forward translations were resolved collaboratively, in line with best practices for cross-cultural adaptation. (c) This reconciled version was then back-translated into English by a native English speaker blinded to the original instrument. (d) Three British midwives reviewed the back-translated version for semantic and conceptual equivalence, and minor modifications were made accordingly.

For cultural adaptation, a cognitive debriefing process was conducted with a sample of midwives to identify potential language issues and to ensure a clear understanding of each item. Following this, seven additional items related to midwives’ professional experience were developed and incorporated into the Greek version of the instrument (Appendix, Tables 8-9). These additions were deemed relevant and appropriate to the context of the study. Such additional questions were included to capture context-specific information pertinent to Greek midwifery practice, particularly in relation to professional experience, training background, and frequency of contact with individuals from the LGBT community. This information was not fully addressed by the original PKSGMH instrument but was considered essential for interpreting responses within the local healthcare context and informing potential educational interventions. The added items were developed in consultation with experts in midwifery and LGBT health and reviewed during the cognitive debriefing process to ensure relevance and clarity. A pilot test was conducted with 10 midwives to assess clarity and comprehension. Feedback focused on the wording of items and instructions, resulting in minor adjustments to improve the readability and usability of the final instrument. This process ensured linguistic accuracy and cultural relevance, aligning the instrument with the specific sociocultural and professional context of midwifery in Greece.

Measures

The survey instrument retained the structure of the original PKSGMH instrument. Specifically, the questionnaire included 81 questions regarding (a) demographics (six questions on age, gender, sexual orientation and marital status), (b) former focused training in LGBT patient care (two questions), (c) attitudes toward LGBT nonpatients (12 questions, five-point Likert scale (5 = strongly agree)), (d) midwives’ attitudes toward LGBT patients (six questions, five-point Likert scale (5 = strongly agree), (e) Sexual and Gender Minority Affirmative Practice Scale (15-question measure with two subscales used a five-point Likert scale), (f) knowledge on LGBT patients (13 true/false questions and nine true/false concerning specific populations; LGBT people), (g) inclusive patient care policy in health facilities (nine true/false questions and two open-ended questions) and (h) professional midwifery experience (seven questions on educational level, years on profession, work environment). The seven questions were designed to capture context-specific information about professional experience, training background, and work environment, elements not covered in the original instrument. These items were reviewed during cognitive debriefing for clarity and relevance. However, because they were context-specific and exploratory, they were not included in the formal reliability testing of the PKSGMH subscales.

This instrument was selected as it is one of the few validated tools specifically developed to measure both cognitive (knowledge-based) and affective (attitudinal and perceptual) domains related to LGBT health, making it particularly valuable for research in healthcare education and professional development. It is grounded in established public health and social psychology frameworks, ensuring theoretical rigour and psychometric robustness [16]. These characteristics make it a suitable and reliable tool for exploring the multifaceted aspects of cultural competence in healthcare provision.

The selection of this tool was further justified by the current gap in the Greek literature regarding standardized instruments capable of assessing midwives’ preparedness to provide inclusive and affirming care to LGBT+ individuals. The PKSGMH not only fills this gap but also provides a means for comparison with international data, facilitating a broader understanding of the cultural and systemic factors that influence healthcare delivery [20,21].

Statistical analysis

Quantitative variables were expressed as mean values (standard deviation) and median (interquartile range), while categorical variables were expressed as absolute and relative frequencies. The Kolmogorov-Smirnov test was used to evaluate whether the normality assumption for a variable was satisfied. The Mann-Whitney test was used to compare continuous variables between two groups. Spearman correlation coefficients (rho) were used to explore the association of two continuous variables. The coefficient is considered very high when it is above 0.9; high when it is 0.7-0.9; moderate when it is 0.5-0.7; low when it is 0.3-0.5; and very low when it is below 0.3 [22]. Intercorrelations regarding construct validity were also evaluated via Spearman correlation coefficients (rho). Internal consistency reliability was determined by the calculation of Cronbach’s α coefficient. Scales with reliabilities equal to or greater than 0.70 were considered acceptable [23]. Multiple linear regression analysis was applied to identify factors associated with the understudy scales, from which regression coefficients (β) and standard errors (SE) were derived. Multiple linear regression analysis was conducted after the dependent variables were logarithmically transformed due to a lack of normal distribution. All reported p-values are two-tailed. Statistical significance was set at p < 0.05, and analyses were conducted using IBM SPSS Statistics for Windows, Version 26 (Released 2018; IBM Corp., Armonk, New York, United States).

## Results

The sample consisted of n = 187 midwives, with a mean age of 41 (SD = 9.9 years). Their characteristics are presented in Table 1. One hundred eighty-four were women (99.5%), and n = 181 were heterosexual (96.8%). Married were 125 (66.8%) of the midwives, and 131 (70.1%) had acquaintances, peers, colleagues, friends, or family who are LGBT. Also, n = 121 (59.9%) midwives were providing care to LGBT patient subgroups and primarily to lesbians (n = 99, 52.9%). Only eight (4.3%) midwives of the sample had received focused training on LGBT patient care, and 140 (76.1%) were aware of gay, lesbian, and bisexual patients in practice. Almost three out of 10 participants (n = 56, 29.9%) got into contact with LGBT people/patients once every six months in the workplace during the last year. The mean years of experience were 15.2 (SD = 9.9 years).

**Table 1:** Sample characteristics

Characteristics	Values	n (%)
Gender	Female	186 (99.5)
	Declined response	1 (0.5)
Sexual orientation	Heterosexual	181 (96.8)
	Homosexual	1 (0.5)
	Bisexual	4 (2.1)
	Declined response	1 (0.5)
Marital status	Married	125 (66.8)
	Partnered	31 (16.6)
	Single	25 (13.4)
	Divorced	6 (3.2)
Frequency of coming into contact with LGBT people/patients in the workplace during the last year	Never	52 (27.8)
	1 time/6 months	56 (29.9)
	1 time/month	24 (12.8)
	1 time/week	10 (5.3)
	Do not know	45 (24.1)

Descriptive measures and reliability indices of study scales are provided in Table 2. The mean total score of attitudes toward gay men, lesbians, bisexuals, and transgender people was 29.94 (SD = 10.49) and toward LGBT patients was 14.24 (SD = 3.21). Additionally, the mean score on the sexual and gender minority affirmative practice scale was 63.52 (SD = 9.79). The mean knowledge score was 9.63 (SD = 2.02). Reliability indexes of all scales were above or close to acceptable levels (≥0.70).

**Table 2:** Descriptive measures and reliability indexes of study scales IQR: interquartile range Note: Greater scores in attitude scores indicate more negative attitudes. Greater scores on the Sexual and Gender Minority Affirmative Practice Scale indicate more positive practice when it comes to LGBT patients. Greater knowledge score indicates greater knowledge

Study scales	Minimum	Maximum	Mean (SD)	Median (IQR)	Cronbach’s alpha
Attitudes toward gay men subscale	3.00	15.00	7.36 (2.69)	7 (6-9)	0.74
Attitudes toward lesbians subscale	3.00	15.00	7.42 (2.75)	7 (6-9)	0.75
Attitudes toward bisexuals subscale	3.00	15.00	7.55 (2.82)	7 (6-9)	0.73
Attitudes toward transgender people subscale	3.00	15.00	7.61 (2.62)	8 (6-9)	0.71
Attitudes toward gay men, lesbians, bisexuals, and transgender people	12.00	60.00	29.94 (10.49)	29 (23-36)	0.94
Attitudes toward lesbian, gay, bisexual, and transgender patients	7.00	26.00	14.24 (3.21)	14 (12-16)	0.70
Sexual and gender minority affirmative practice scale	30.00	75.00	63.52 (9.79)	63 (58-73)	0.93
Knowledge score	4.00	13.00	9.63 (2.02)	10 (8-11)	0.94

More than 50% of midwives responded correctly to true/false questions about the prevalence of depression and anxiety (n = 128, 68.4%), having less access to healthcare services (n = 95, 50.8%), the prevalence of sexually transmitted infections (n = 123, 65.8%), and the prevalence of physical and sexual trauma in LGBT patients (n = 133, 71.1%). However, less than 50% of them answered correctly on issues regarding the higher prevalence of tobacco (n = 79, 42.2%), alcohol (n = 77, 41.2%) and substance use (n = 87, 46.5%), increased prevalence of obesity/overweight (n = 42, 22.5%), and greater risk for chronic disease (n = 72, 38.5%) in the LGBT community.

Dimensions of the questionnaire were compared according to midwives’ characteristics in terms of discriminant validity (i.e., it was expected that those that had acquaintance, peers, colleagues, friends, or family who are LGBT, were primary care providers providing care to LGBT patient subgroups, had received focused training on LGBT patient care would have better attitude scores. Significant and positive correlations among attitude scales toward LGBT people were found (Table 3). Specifically, a more negative attitude toward LGBT people in general was significantly associated with more negative attitudes toward LGBT patients. Furthermore, a more negative attitude toward LGBT individuals/patients was significantly correlated with reduced practice in providing care for LGBT patients. Greater knowledge was significantly associated with a less negative attitude toward LGBT people/patients and better practice when it comes to LGBT patients.

**Table 3:** Spearman correlation coefficients (rho) among understudy scales Note: All coefficients were significant at p < 0.001

	Attitudes toward lesbians subscale	Attitudes toward bisexuals subscale	Attitudes toward transgender people subscale	Attitudes toward gay men, lesbians, bisexuals, and transgender people	Attitudes toward lesbian, gay, bisexual, and transgender patients	Sexual and gender minority affirmative practice scale	Knowledge score
Attitudes toward gay men subscale	0.99	0.89	0.85	0.98	0.41	-0.56	-0.49
Attitudes toward lesbians subscale	1.00	0.91	0.84	0.98	0.41	-0.56	-0.49
Attitudes toward bisexuals subscale		1.00	0.84	0.95	0.45	-0.53	-0.51
Attitudes toward transgender people subscale			1.00	0.91	0.45	-0.53	-0.50
Attitudes toward gay men, lesbians, bisexuals, and transgender people				1.00	0.44	-0.57	-0.52
Attitudes toward lesbian, gay, bisexual, and transgender patients					1.00	-0.34	-0.24
Sexual and Gender Minority Affirmative Practice Scale						1.00	0.47

Married/partnered participants had significantly more negative attitude scales toward LGBT people, as well as those who had no LGBT acquaintances, peers, colleagues, friends, or family members, and those who had not provided care to LGBT patient subgroups (Table 4). On the contrary, midwives who had received focused training on LGBT patient care had significantly less negative attitude scales toward LGBT people. Greater age and more experience were significantly associated with more negative attitude scales toward LGBT people (Table 5). In contrast, a greater frequency of coming into contact with LGBT people/patients in the workplace during the past year was significantly associated with less negative attitude scales toward LGBT people.

**Table 4:** Association of attitude scores toward LGBT people with midwives’ characteristics LGBT: lesbian, gay, bisexual, and transgender ^+^p-value from the Mann-Whitney test

	Attitudes toward gay men subscale			Attitudes toward lesbians subscale			Attitudes toward bisexuals subscale			Attitudes toward transgender people subscale			Attitudes toward gay men, lesbians, bisexuals and transgender people		
	Mean (SD)	Median (IQR)	P^+^	Mean (SD)	Median (IQR)	P^+^	Mean (SD)	Median (IQR)	P^+^	Mean (SD)	Median (IQR)	P^+^	Mean (SD)	Median (IQR)	P^+^
Married/partnered															
No	6.16 (2.75)	7 (3-8)	0.011	6.26 (2.83)	7 (3-8)	0.013	6.55 (3.18)	7 (3-8)	0.025	6.26 (2.38)	6 (4-8)	0.002	25.23 (10.63)	26 (15-32)	0.009
Yes	7.6 (2.62)	7 (6-9)		7.65 (2.68)	7.5 (6-9)		7.74 (2.7)	8 (6-9)		7.88 (2.59)	8 (6-9)		30.88 (10.24)	30 (24-36.5)	
Had acquaintances, peers, colleagues, friends, or family who are LGBT															
No	8.57 (2.72)	9 (7-10)	<0.001	8.61 (2.78)	9 (7-10)	<0.001	8.79 (2.77)	9 (7-10)	<0.001	8.96 (2.52)	9 (8-10)	<0.001	34.93 (10.35)	36 (28.5-39)	<0.001
Yes	6.85 (2.51)	7 (5-9)		6.91 (2.58)	7 (5-9)		7.02 (2.67)	7 (5-9)		7.04 (2.45)	7 (6-9)		27.81 (9.84)	28 (21-34)	
Primary care providers providing care to LGBT patient subgroups															
No	7.75 (2.81)	7 (6-9)	0.200	7.85 (2.91)	8 (6-9)	0.145	8.21 (2.91)	8 (6-10)	0.010	8.37 (2.8)	9 (7-10)	<0.001	32.19 (10.96)	32 (24-38)	0.037
Yes	7.11 (2.58)	7 (5-9)		7.13 (2.61)	7 (5-9)		7.1 (2.67)	7 (5-9)		7.11 (2.37)	7 (6-9)		28.44 (9.93)	28.5 (21.5-35)	
Received focused training on LGBT patient care															
No	7.51 (2.63)	7 (6-9)	<0.001	7.56 (2.7)	7 (6-9)	<0.001	7.68 (2.78)	8 (6-9)	0.002	7.76 (2.56)	8 (6-9)	<0.001	30.51 (10.28)	30 (24-36)	<0.001
Yes	4 (1.51)	3 (3-5)		4.25 (1.75)	3.5 (3-5)		4.63 (2)	4 (3-6)		4.38 (1.77)	3.5 (3-6)		17.25 (6.58)	15 (12-21)	
Aware of gay, lesbian, and bisexual patients in practice															
No	7.39 (2.83)	7 (5-9)	0.821	7.45 (2.87)	8 (5-9)	0.751	7.61 (2.84)	8 (6-9)	0.675	7.73 (2.8)	8 (6-9)	0.678	30.18 (10.88)	30 (22.5-36.5)	0.676
Yes	7.31 (2.65)	7 (6-9)		7.36 (2.71)	7 (6-9)		7.46 (2.81)	7 (6-9)		7.54 (2.57)	7 (6-9)		29.67 (10.41)	28.5 (23.5-36)	
Aware of transgender patients in practice															
No	7.08 (2.42)	7 (5-9)	0.578	7.08 (2.4)	7.5 (5-9)	0.522	7.2 (2.27)	8 (6-9)	0.755	7.23 (2.26)	7 (6-9)	0.384	28.58 (8.81)	30 (22.5-36)	0.601
Yes	7.57 (2.76)	7 (6-9)		7.64 (2.84)	7 (6-9)		7.69 (2.95)	7 (6-9)		7.78 (2.71)	8 (6-9)		30.68 (10.93)	29 (24-37)	

For LGBT patients in particular (Table 5), midwives with more negative attitudes were found to be those who had no LGBT acquaintances, peers, colleagues, friends, or family, those who had not provided care to LGBT patient subgroups, and those who had not received specialized training on LGBT patient care. Moreover, greater age and more experience were significantly associated with more negative attitude scales toward LGBT patients (rho = 0.19, p = 0.01; and rho = 0.25, p = 0.001, respectively).

**Table 5:** Association of attitude scores toward LGBT patients with midwives’ characteristics LGBT: lesbian, gay, bisexual, and transgender; ^+^p-value from Mann-Whitney test

	Attitudes toward lesbian, gay, bisexual, and transgender patients		
	Mean (SD)	Median (IQR)	P^+^
Married/partnered			
No	13.19 (2.5)	13 (12-15)	0.090
Yes	14.45 (3.3)	14 (12-16)	
Had acquaintances, peers, colleagues, friends, or family who are LGBT			
No	15.64 (3.75)	15 (13-16)	<0.001
Yes	13.64 (2.75)	13 (12-15)	
Primary care providers providing care to LGBT patient subgroups			
No	15.36 (3.49)	15 (13-16)	<0.001
Yes	13.49 (2.78)	13 (12-15)	
Received focused training on LGBT patient care			
No	14.36 (3.19)	14 (12-16)	0.017
Yes	11.5 (2.45)	13 (9-13)	
Aware of gay, lesbian, and bisexual patients in practice			
No	14.07 (2.49)	14 (12.5-16)	0.819
Yes	14.26 (3.43)	14 (12-16)	
Aware of transgender patients in practice			
No	14.28 (2.31)	14 (13-16)	0.478
Yes	14.28 (3.53)	14 (12-16)	

Similarly, significantly lower knowledge and less positive practices toward LGBT patients were observed among married/partnered participants, those without LGBT acquaintances, whether peers, colleagues, friends, or family, and those lacking experience in providing care to LGBT patient subgroups. Greater age and more years of professional experience were significantly associated with less positive practice toward LGBT patients and less knowledge. On the contrary, midwives who had received focused training on LGBT patient care had significantly greater knowledge. Finally, a higher frequency of contact with LGBT individuals/patients in the workplace over the past year was significantly associated with greater knowledge and more positive practices toward LGBT patients (Tables 6-7).

**Table 6:** Association of Sexual and Gender Minority Affirmative Practice Scale with midwives’ characteristics LGBT: lesbian, gay, bisexual, and transgender ^+^p-value from Mann-Whitney test

	Sexual and Gender Minority Affirmative Practice Scale		
	Mean (SD)	Median (IQR)	P^+^
Married/partnered			
No	68.13 (8.35)	72 (62-75)	0.002
Yes	62.61 (9.81)	61.5 (57.5-72)	
Had acquaintances, peers, colleagues, friends, or family who are LGBT			
No	58.39 (10.44)	60 (54.5-64)	<0.001
Yes	65.72 (8.64)	68 (59-74)	
Primary care providers providing care to LGBT patient subgroups			
No	61.56 (11.19)	60 (55-72)	0.109
Yes	64.84 (8.52)	64 (59-73)	
Received focused training on LGBT patient care			
No	63.09 (9.77)	62 (58-72)	0.003
Yes	73.13 (2.64)	73.5 (73-75)	
Aware of gay, lesbian, and bisexual patients in practice			
No	64.41 (9.51)	67 (58-73)	0.716
Yes	63.47 (9.83)	63 (58-73)	
Aware of transgender patients in practice			
No	64.95 (9.24)	67 (60-73)	0.317
Yes	63 (10.17)	62.5 (57-73)	

**Table 7:** Association of knowledge score with midwives’ characteristics LGBT: lesbian, gay, bisexual, and transgender; IQR: interquartile range ^+^p-value from Mann-Whitney test

	Knowledge score		
	Mean (SD)	Median (IQR)	P^+^
Married/partnered			
No	10.19 (2.06)	11 (9-12)	0.046
Yes	9.52 (2.00)	10 (8-11)	
Had acquaintances, peers, colleagues, friends, or family who are LGBT			
No	8.48 (1.92)	9 (7-10)	<0.001
Yes	10.12 (1.87)	11 (9-12)	
Primary care providers providing care to LGBT patient subgroups			
No	9.25 (2.19)	10 (7-11)	0.050
Yes	9.88 (1.87)	10 (9-11.5)	
Received focused training on LGBT patient care			
No	9.58 (1.99)	10 (8-11)	0.020
Yes	10.88 (2.42)	12 (11-12)	
Aware of gay, lesbian, and bisexual patients in practice			
No	9.70 (1.77)	10 (8.5-11)	0.849
Yes	9.67 (2.07)	10 (8-11)	
Aware of transgender patients in practice			
No	9.75 (1.84)	10 (8-11)	0.749
Yes	9.58 (2.06)	10 (8-11)	

After multiple linear regression analyses, it was found that having acquaintance, peers, colleagues, friends, or family who are LGBT was associated with significantly more positive attitudes toward gay men (β = -0.068; p = 0.031), toward bisexual (β = -0.067; p = 0.047), toward transgender (β = -0.086; p = 0.004), and more positive overall attitudes toward LGBT people (β = -0.070; p = 0.020). Additionally, having acquaintances, peers, colleagues, friends, or family who are LGBT was associated with significantly more positive attitudes toward this group (β = 0.042; p = 0.003) and with greater knowledge on the subject (β = 0.073; p < 0.001). Furthermore, having received focused training on LGBT patient care was significantly associated with more positive attitudes toward LGBT people (β ranged from -0.267 to -0.181; p < 0.05) and LGBT patients (β = -0.111; p = 0.015). Participants who were primary care providers providing care to LGBT patient subgroups had significantly more positive attitudes toward LGBT patients (β = -0.065; p = 0.002), whereas having greater working experience was significantly associated with less positive attitudes toward LGBT patients (β = 0.005; p = 0.003). Being aware of gay, lesbian, and bisexual patients in practice was significantly associated with significantly worse practice toward this group (β = -0.052; p = 0.047), while greater frequency of coming into contact with LGBTI+ people/patients in workplace during last year was significantly associated with more good practice toward this group (β = 0.026; p = 0.002). Being aware of transgender patients in practice was significantly associated with significantly fewer positive attitudes toward transgender (β = 0.112; p = 0.025). 

## Discussion

The Greek version of the PKSGMH instrument demonstrated sound psychometric properties, yielding reliable and valid measures of midwives’ knowledge, attitudes, and perceived institutional barriers to providing LGBT-affirming care. However, our findings must be interpreted within the context of several methodological considerations and compared critically to existing literature.

Knowledge and LGBT-focused training

Midwives in our sample demonstrated moderate to high awareness of key health issues affecting LGBT individuals, including mental health concerns (such as depression and anxiety), sexually transmitted infections, and experiences of physical or sexual trauma. However, substantial knowledge gaps and persistent misconceptions were evident regarding the increased prevalence of substance use (including tobacco and alcohol), overweight/obesity, and chronic disease risk among LGBT populations. These findings are consistent with prior studies involving other healthcare professional groups [16,20], highlighting a widespread need for more comprehensive education in this area.

Although only 4.3% of our participants (n = 8) reported having received formal LGBT health training, which is significantly lower than the 15% reported in international studies [15,16,24], this subgroup nonetheless scored significantly higher on both the knowledge and affirmative practice scales (p < 0.05). While this small number limited our ability to perform detailed subgroup analysis, it suggests that even minimal formal training may be associated with improved competence. On the other hand, it points out the lack of structured continuing education in this field in Greece.

Interestingly, despite the low formal training rate, many midwives reported exposure to LGBT individuals in their personal and professional lives. Specifically, 70.1% (n = 131) had a friend or family member who identified as LGBT, and 59.9% (n = 112) had provided care to LGBT patients. This exposure may have contributed to the acquisition of informal knowledge through self-directed learning and professional experience. These findings highlight the importance of lived experience and personal connections as informal educational channels, although they are not a substitute for structured, evidence-based training.

Moreover, only 13 (6.9%) participants agreed that their undergraduate training adequately prepared them to address the needs of the LGBT population, a proportion significantly smaller than the 29% reported in the original study [15]. Continuing education and training of healthcare professionals are essential for ensuring high-quality patient care and promoting health equity across diverse populations [25-27]. In certain countries, relevant authorities advocate implementing educational programs in healthcare institutions that address the distinct needs of the LGBT community and aim for culturally competent healthcare [28]. Furthermore, research data highlight the educational needs of midwives to feel more confident in providing better care to LGBT individuals [29]. It is concerning that Greece currently lacks a coordinated national strategy for training healthcare professionals in LGBT health. While this remains a significant gap, recent curricular developments offer some promise. The three public university midwifery departments in Greece have begun incorporating educational modules addressing the health needs of vulnerable populations, including LGBT individuals. However, the real-world impact of these curricular changes is likely to become evident only gradually, as future cohorts enter clinical practice and educational interventions are evaluated for effectiveness.

Attitudes toward LGBT people and patients

Overall, midwives’ attitudes toward LGBT individuals and patients ranged from neutral to moderately positive, consistent with findings in allied health professions in Mediterranean contexts, such as Italy and Spain, where comparable surveys report mean attitude scores between 3.5 and 4.2 on five-point Likert scales [30,31]. In our study, professionals with many years of experience tended to have less positive attitudes toward LGBT. This finding is supported by previous studies, which have linked generational differences in the openness of healthcare providers to the era in which they were trained and to prevailing social attitudes at that time. 

Increased knowledge regarding LGBT health was significantly associated with a less negative attitude toward LGBT individuals/patients, as well as better practice when it comes to LGBT patients. These findings align with those of previous studies [32-34] and a recent meta-analysis of healthcare provider bias [35] but are contrary to the conclusions of a more recent review [36]. Furthermore, characteristics such as younger age and having a friend or a family member who identified as LGBT were linked to more positive attitudes toward LGBT patients, findings consistent with the results of other studies [37,38].

Little is known about the direct impact of healthcare providers’ negative attitudes on healthcare outcomes. It is suggested that perceived negative attitudes of healthcare providers are a major causative factor of stigma and marginalization that leads to health disparities for LGBT individuals [36]. On the other hand, healthcare professionals may have negative attitudes toward LGBT patients due to knowledge or training deficits, and this can ultimately lead to persistent mistreatment of these patients in healthcare systems [39]. Those aspects underscore the importance of studying and trying to improve healthcare providers’ attitudes toward LGBT individuals.

Recent literature indicates a shift in registered nurses’ attitudes toward working with sexual minorities, reflecting the broader changes in social norms and policy reforms across several countries [40]. In a more recent systematic review exploring the attitudes of nurses and midwives toward LGBT people, authors described the broad spectrum of attitudes recorded as the "rainbow of attitudes." Moreover, the authors suggest that heteronormativity shapes healthcare staff attitudes in heteronormative environments, with nurses and midwives reinforcing this culture within healthcare settings, underlying that midwives should be cognizant of the attitudes and beliefs that may influence their care of LGBT patients [41].

Strengths and limitations of the study

This study bears several limitations. One is the moderate sample size; however, it exceeds that of Rowe’s original study and is considered adequate for an exploratory investigation. A key challenge in Greece is the absence of a centralized national registry of midwives, which complicates efforts to obtain a complete and current estimate of all practising professionals across different sectors. According to the most recent available data, Greece has approximately 0.27 midwives per 1,000 people. With a national population of around 10.7 million, this translates to an estimated 2,890 practicing midwives. Our sample of 187 participants therefore represents roughly 6.5%-7% of the national midwifery workforce, which is a relatively strong representation for a pilot study of this nature [42].

Secondly, the use of an online survey methodology may have introduced sampling bias, potentially favoring participants with higher digital literacy and access. As such, older or less technologically engaged midwives may be underrepresented. Additionally, the convenience sampling approach carries a risk of self-selection bias, whereby individuals with more positive attitudes toward LGBT care may have been more inclined to participate. Despite these limitations, our sample included a heterogeneous mix of participants across age groups, geographic regions, and workplace settings, enhancing the diversity of perspectives captured. Notably, a stratified sampling strategy was not feasible due to the absence of a national registry or centralized database of practising midwives in Greece. In light of this constraint, we employed a pragmatic recruitment approach by disseminating the survey not only through social media but also via the official communication channels of all nine Greek midwifery professional bodies. This was done intentionally to maximize outreach, improve representation across the profession, and reduce the biases typically associated with general online recruitment. Conversely, some responses, particularly on the Sexual and Gender Minority Affirmative Practice Scale, may have been affected by social desirability bias, with midwives potentially adjusting their answers to align with the expectation of treating all patients equally. One limitation of this study is the absence of a direct comparison between the Greek version of the instrument and the original version within the same study population. This limitation restricts the ability to fully assess cross-cultural equivalence; future studies may consider administering both versions to bilingual populations to explore this aspect of validity further.

One of the key strengths of this study is the inclusion of a diverse sample of certified professional midwives from across Greece, encompassing a broad spectrum of ages, workplace settings, years of clinical experience, and geographic regions. This heterogeneity enhances the generalizability of the findings and facilitates a more comprehensive understanding of current midwifery practices about LGBT health. Additionally, the questionnaire employed included a wide range of items assessing both general and specific knowledge about LGBT patients, offering a nuanced view of participants’ baseline understanding. Notably, this is the first study in Greece to evaluate midwives’ knowledge and attitudes toward LGBT health using a validated and culturally adapted instrument.

Policy and educational implications

Our findings highlight the imperative for structured, evidence-based LGBT content in both undergraduate midwifery curricula and continuing professional development in delivering culturally responsive midwifery care to patients with minoritized sexual and gender identities.

Preregistration curricular integration is necessary to embed mandatory modules on LGBT health across all midwifery programs, emphasising intersectionality and real-world case studies. Educational sessions incorporating both didactic and interactive components could be crucial in improving clinical preparedness and overcoming existing barriers to training.

Continuing professional development for midwives is also vital in addressing knowledge gaps, particularly regarding misconceptions about the higher prevalence of substance use (including tobacco and alcohol), obesity/overweight, and an increased risk for chronic diseases among these populations. Accredited workshops and online courses should be developed, ideally in collaboration with LGBT community organizations, to address identified knowledge gaps.

Action is needed to overcome key barriers to implementation, such as a lack of institutional prioritization and curriculum overload, through coordinated efforts between the Hellenic Ministry of Health and academic institutions. These stakeholders should work jointly to develop a phased strategy for integrating LGBT health content into midwifery education and continuing professional development. A national training framework should be established to ensure consistent standards, monitor compliance, and guide the long-term implementation and sustainability of training initiatives. This initiative must be reinforced by the adoption of clear nondiscrimination policies and inclusive care guidelines within healthcare facilities, which professional bodies and regulatory agencies should support.

Future research directions

Further research is necessary to explore the effectiveness of various training programs in enhancing midwives’ competence in LGBT issues and to investigate the long-term outcomes, such as healthcare access, for LGBT patients receiving care from culturally competent midwives. Future studies should also examine patient-centered outcomes by investigating how midwives’ cultural competence affects the satisfaction, healthcare access, and perinatal outcomes of LGBT patients using mixed-methods designs. Furthermore, comparative analyses could be conducted in conjunction with cross-national research involving countries with similar healthcare structures to contextualize Greek findings within broader European frameworks.

## Conclusions

The Greek version of the PKSGMH instrument demonstrated strong reliability and validity, confirming its suitability for assessing knowledge, perceptions, and attitudes toward LGBT individuals among midwives and other healthcare professionals. Its application can support future research and practice not only in Greece but also in countries with comparable cultural and healthcare contexts. Our findings indicate that Greek midwives possess moderate to high levels of knowledge and generally neutral to positive attitudes toward LGBT individuals and patients. However, detailed analysis revealed notable knowledge gaps, misconceptions, and self-reported limitations in clinical preparedness. Notably, the small proportion of participants who had received postgraduate training on LGBT health scored significantly higher across all domains of the instrument. These results emphasize the urgent need for strategic planning in midwifery continuing education, particularly the inclusion of targeted, evidence-based content focused on LGBT health. Ultimately, this study contributes valuable insights into the existing gaps and institutional challenges that must be addressed to ensure equitable, person-centered midwifery care for sexual and gender minority populations.

## Appendices

**Table 8:** The Greek version of the PKSGMH instrument used for the study

Εργαλείο αξιολόγησης των αντιλήψεων και των γνώσεων για την υγεία των Σεξουαλικών και Φυλετικών Μειονοτήτων						
1.	Πόσα χρόνια εργάζεστε ως επαγγελματίας μαία/ μαιευτής;					
2.	Φύλο					
	α. Άνδρας β. Γυναίκα γ. Τρανσέξουαλ (transgender) δ. Προτιμώ να μην απαντήσω					
3.	Σεξουαλικός προσανατολισμός					
	α. Ετερόφυλος/-η β. Ομοφυλόφυλος/-η γ. Αμφιφυλόφιλος (bisexual) δ. Προτιμώ να μην απαντήσω					
4.	Οικογενειακή κατάσταση					
	α. Παντρεμένος/-η β. Σε μόνιμη σχέση γ. Ελεύθερος/η δ. Σε διάσταση ε. Χήρος/-α					
5.	Έχετε γνωστούς, συνομηλίκους, συναδέλφους, φίλους ή μέλη της οικογένειάς σας που να ανήκουν στη ΛΟΑΤΚΙ κοινότητα;					
	α. Ναι β. Όχι					
6.	Παρέχετε πρωτοβάθμια φροντίδα σε άτομα/ ασθενείς που ανήκουν σε σεξουαλική μειονότητα ή στη ΛΟΑΤΚΙ κοινότητα; Επιλέξτε όσα ισχύουν:					
	α. Γκέι γυναίκες β. Γκέι άντρες γ. Αμφιφυλόφιλους (bisexual) δ. Τρανσέξουαλ (transgender)					
7.	Έχετε λάβει εκπαίδευση επικεντρωμένη στη φροντίδα των ΛΟΑΤΚΙ ατόμων/ασθενών;					
	α. Ναι β. Όχι					
8.	Εάν, ναι, πού και πότε λάβατε εκπαίδευση σχετικά με τη φροντίδα των ΛΟΑΤΚΙ ατόμων/ ασθενών;					
	Πού:_________________________________________________________________					
	Πότε: __/__/__ (ηη/μμ/εε)					
Ακολουθούν ορισμένες ερωτήσεις σχετικά με τη γνώμη σας για τους ομοφυλόφιλους άντρες και γυναίκες και τα αμφιφυλόφυλα (bisexual) και τρανσέξουαλ (transgender) άτομα. Παρακαλώ, διαβάστε κάθε δήλωση και σημειώστε τον βαθμό συμφωνίας ή διαφωνίας σας στην κλίμακα που ακολουθεί. Όλες οι απαντήσεις θα διατηρηθούν εμπιστευτικές.						
	Στάση απέναντι στους γκέι άνδρες. Επιλέξτε μια απάντηση ανά σειρά					
9.	Το σεξ μεταξύ δύο ανδρών είναι απλά λάθος	Διαφωνώ έντονα	Διαφωνώ	Ουδέτερος/-η ούτε διαφωνώ/ούτε συμφωνώ	Συμφωνώ	Συμφωνώ έντονα
10.	Θεωρώ ότι οι ομοφυλόφιλοι άνδρες είναι αηδιαστικοί	Διαφωνώ έντονα	Διαφωνώ	Ουδέτερος/-η ούτε διαφωνώ/ούτε συμφωνώ	Συμφωνώ	Συμφωνώ έντονα
11.	Η ανδρική ομοφυλοφιλία αποτελεί φυσική έκφραση της σεξουαλικότητας των ανδρών	Διαφωνώ έντονα	Διαφωνώ	Ουδέτερος/-η ούτε διαφωνώ/ούτε συμφωνώ	Συμφωνώ	Συμφωνώ έντονα
	Στάση απέναντι στις γκέι γυναίκες. Επιλέξτε μια απάντηση ανά σειρά					
12.	Το σεξ μεταξύ δύο γυναικών είναι απλά λάθος	Διαφωνώ έντονα	Διαφωνώ	Ουδέτερος/-η ούτε διαφωνώ/ούτε συμφωνώ	Συμφωνώ	Συμφωνώ έντονα
13.	Θεωρώ ότι οι ομοφυλόφιλες γυναίκες είναι αηδιαστικές	Διαφωνώ έντονα	Διαφωνώ	Ουδέτερος/-η ούτε διαφωνώ/ούτε συμφωνώ	Συμφωνώ	Συμφωνώ έντονα
14.	Η γυναικεία ομοφυλοφιλία αποτελεί φυσική έκφραση της σεξουαλικότητας των γυναικών	Διαφωνώ έντονα	Διαφωνώ	Ουδέτερος/-η ούτε διαφωνώ/ούτε συμφωνώ	Συμφωνώ	Συμφωνώ έντονα
	Στάση απέναντι στα αμφιφυλόφυλα (bisexual) άτομα. Επιλέξτε μια απάντηση ανά σειρά					
15.	Το να κάνεις σεξ τόσο με άνδρες όσο και με γυναίκες είναι απλά λάθος	Διαφωνώ έντονα	Διαφωνώ	Ουδέτερος/-η ούτε διαφωνώ/ούτε συμφωνώ	Συμφωνώ	Συμφωνώ έντονα
16.	Θεωρώ ότι τα αμφιφυλόφιλα άτομα (bisexuals) είναι αηδιαστικά	Διαφωνώ έντονα	Διαφωνώ	Ουδέτερος/-η ούτε διαφωνώ/ούτε συμφωνώ	Συμφωνώ	Συμφωνώ έντονα
17.	Η αμφιφυλοφιλία (bisexuality) αποτελεί φυσική έκφραση της σεξουαλικότητας των ανδρών και των γυναικών	Διαφωνώ έντονα	Διαφωνώ	Ουδέτερος/-η ούτε διαφωνώ/ούτε συμφωνώ	Συμφωνώ	Συμφωνώ έντονα
	Στάση απέναντι στα τρανσέξουαλ (transgender) άτομα. Επιλέξτε μια απάντηση ανά σειρά					
18.	Το να μη συμβαδίζει η ταυτότητα φύλου (άνδρας ή γυναίκα) ενός ατόμου με το βιολογικό του φύλο/φύλο που του αποδόθηκε στη γέννηση (άρρεν ή θήλυ) είναι απλά λάθος	Διαφωνώ έντονα	Διαφωνώ	Ουδέτερος/-η ούτε διαφωνώ/ούτε συμφωνώ	Συμφωνώ	Συμφωνώ έντονα
19.	Θεωρώ ότι τα τρανσέξουαλ άτομα είναι αηδιαστικά	Διαφωνώ έντονα	Διαφωνώ	Ουδέτερος/-η ούτε διαφωνώ/ούτε συμφωνώ	Συμφωνώ	Συμφωνώ έντονα
20.	Το να είσαι τρανσέξουαλ αποτελεί φυσική έκφραση της ταυτότητας φύλου σε άνδρες και γυναίκες	Διαφωνώ έντονα	Διαφωνώ	Ουδέτερος/-η ούτε διαφωνώ/ούτε συμφωνώ	Συμφωνώ	Συμφωνώ έντονα
Οι επόμενες ερωτήσεις αφορούν τα συναισθήματά σας σχετικά με την παροχή υγειονομικής φροντίδα σε ασθενείς/ άτομα ΛΟΑΤΚΙ. Παρακαλούμε διαβάστε κάθε δήλωση και σημειώστε το επίπεδο συμφωνίας ή διαφωνίας σας στην παρακάτω κλίμακα. Όλες οι απαντήσεις διατηρούνται εμπιστευτικές.						
21.	Θα προτιμούσα να μην παρέχω φροντίδα σε ΛΟΑΤΚΙ άτομα/ ασθενείς	Διαφωνώ έντονα	Διαφωνώ	Ουδέτερος/-η ούτε διαφωνώ/ούτε συμφωνώ	Συμφωνώ	Συμφωνώ έντονα
22.	Θα αρνιόμουν τη φροντίδα σε ένα ΛΟΑΤΚΙ άτομο/ ασθενή εάν γνώριζα ότι αυτοπροσδιορί ζεται ως ΛΟΑΤΚΙ	Διαφωνώ έντονα	Διαφωνώ	Ουδέτερος/-η ούτε διαφωνώ/ούτε συμφωνώ	Συμφωνώ	Συμφωνώ έντονα
23.	Αισθάνομαι ικανός/-ή να παρέχω φροντίδα σε ΛΟΑΤΚΙ άτομα/ασθενείς	Διαφωνώ έντονα	Διαφωνώ	Ουδέτερος/-η ούτε διαφωνώ/ούτε συμφωνώ	Συμφωνώ	Συμφωνώ έντονα
24.	Τα ΛΟΑΤΚΙ άτομα/ ασθενείς δεν έχουν ιδιαίτερες ανάγκες υγείας	Διαφωνώ έντονα	Διαφωνώ	Ουδέτερος/-η ούτε διαφωνώ/ούτε συμφωνώ	Συμφωνώ	Συμφωνώ έντονα
25.	Νιώθω ότι θα μπορούσα να μιλήσω σε ασθενή/ άτομο που προσδιορίζεται ως ΛΟΑΤΚΙ+ με ευαίσθητο και κατάλληλο τρόπο	Διαφωνώ έντονα	Διαφωνώ	Ουδέτερος/-η ούτε διαφωνώ/ούτε συμφωνώ	Συμφωνώ	Συμφωνώ έντονα
26.	Πιστεύω ότι η βασική/ προπτυχιακή μου εκπαίδευση καλύπτει επαρκώς τις ανάγκες υγείας του ΛΟΑΤΚΙ+ πληθυσμού	Διαφωνώ έντονα	Διαφωνώ	Ουδέτερος/-η ούτε διαφωνώ/ούτε συμφωνώ	Συμφωνώ	Συμφωνώ έντονα
Παρακαλώ, σημειώστε εάν πιστεύετε ότι οι παρακάτω δηλώσεις είναι σωστές ή λάθος.						
27.	Οι όροι «φύλο/ βιολογικό φύλο/ sex» και «γένος/ κοινωνικό φύλο/ gender» έχουν την ίδια σημασία				Σωστό	Λάθος
28.	Τα περισσότερα ομοφυλόφιλα άτομα θέλουν να ανήκουν στο αντίθετο φύλο				Σωστό	Λάθος
29.	Οι ομοφυλόφιλοι άνδρες πάντα συμπεριφέρονται και ντύνονται θηλυπρεπώς				Σωστό	Λάθος
30.	Οι ομοφυλόφιλοι άνδρες είναι πιο πιθανό να υπάρξουν θύματα βίαιων εγκλημάτων, σε σύγκριση με τον γενικό πληθυσμό				Σωστό	Λάθος
31.	Τα αμφιφυλόφιλα άτομα (bisexuals) τελικά θα «αποκαλυφθούν» (“coming out”) ως ομοφυλόφιλα				Σωστό	Λάθος
32.	Η αμφιφυλοφιλική συμπεριφορά είναι συχνά απλώς μια «κραυγή για προσοχή» (a cry for attention)				Σωστό	Λάθος
33.	Για να θεωρηθεί κάποιο άτομο τρανσέξουαλ, θα πρέπει να έχει κάνει χειρουργική διόρθωση φύλου				Σωστό	Λάθος
34.	Οι γυναίκες τρανσέξουαλ (από άνδρα-> σε γυναίκα) ελκύονται πάντα από άτομα με ανδρικά γεννητικά όργανα				Σωστό	Λάθος
35.	Σε ένα τρανσέξουαλ άτομο θα πρέπει να απευθυνόμαστε χρησιμοποιώντας τις αντωνυμίες του προτιμώμενου φύλου και όχι του βιολογικού φύλου				Σωστό	Λάθος
36.	Οι ομοφυλόφιλες γυναίκες ντύνονται και συμπεριφέρονται πάντα ανδροπρεπώς				Σωστό	Λάθος
37.	Οι ΛΟΑΤΚΙ ασθενείς/ τα ΛΟΑΤΚΙ άτομα δεν αναζητούν ιατρική περίθαλψη τόσο νωρίς όσο οι ετεροφυλόφιλοι λόγω του φόβου των διακρίσεων				Σωστό	Λάθος
38.	Οι περισσότεροι επαγγελματίες υγείας αυτόματα υποθέτουν ότι ο ασθενής τους είναι ετεροφυλόφιλος, εάν δεν έχουν αναφερθεί ειδικά στον σεξουαλικό προσανατολισμό				Σωστό	Λάθος
39.	Οι ΛΟΑΤΚΙ ασθενείς/ τα ΛΟΑΤΚΙ άτομα μπορεί να παρουσιάζουν σημάδια κατάθλιψης λόγω έλλειψης κοινωνικής αποδοχής				Σωστό	Λάθος
Παρακαλώ, σημειώστε εάν πιστεύετε ότι οι παρακάτω δηλώσεις είναι σωστές ή λάθος. Εάν είναι σωστές, παρακαλώ σημειώστε για ποιες υποομάδες είναι σωστή η δήλωση (μπορείτε να σημειώσετε περισσότερες από μία υποομάδα).						
40.	Οι σεξουαλικές μειονότητες εμφανίζουν μεγαλύτερη συχνότητα κατάθλιψης και άγχους από ό,τι οι ετεροφυλόφιλοι Γκέι γυναίκες o Γκέι άντρες o Αμφιφυλόφιλοι o Τρανσέξουαλς o				Σωστό	Λάθος
41.	Οι σεξουαλικές μειονότητες έχουν υψηλότερα ποσοστά χρήσης αλκοόλ και προβλημάτων λόγω της χρήσης αλκοόλ από ό,τι οι ετεροφυλόφιλοι και οι σεξουαλικές πλειοψηφίες (gender majorities). Γκέι γυναίκες o Γκέι άντρες o Αμφιφυλόφιλοι o Τρανσέξουαλς o				Σωστό	Λάθος
42.	Οι σεξουαλικές μειονότητες έχουν δύο φορές περισσότερες πιθανότητες να κάνουν χρήση καπνού από ό,τι οι ετεροφυλόφιλοι & οι σεξουαλικές πλειοψηφίες (gender majorities). Γκέι γυναίκες o Γκέι άντρες o Αμφιφυλόφιλοι o Τρανσέξουαλς o				Σωστό	Λάθος
43.	Οι σεξουαλικές μειονότητες αναφέρουν υψηλότερη χρήση ουσιών από ό,τι οι ετεροφυλόφιλοι & οι σεξουαλικές πλειοψηφίες (gender majorities). Γκέι γυναίκες o Γκέι άντρες o Αμφιφυλόφιλοι o Τρανσέξουαλς o				Σωστό	Λάθος
44.	Οι σεξουαλικές μειονότητες έχουν λιγότερη πρόσβαση στην αναζήτηση υπηρεσιών υγειονομικής περίθαλψης από ό,τι οι ετεροφυλόφιλοι & οι σεξουαλικές πλειοψηφίες (gender majorities). Γκέι γυναίκες o Γκέι άντρες o Αμφιφυλόφιλοι o Τρανσέξουαλς o				Σωστό	Λάθος
45.	Οι σεξουαλικές μειονότητες έχουν υψηλότερα ποσοστά παχυσαρκίας/αυξημένου βάρους από ό,τι οι ετεροφυλόφιλοι & οι σεξουαλικές πλειοψηφίες (gender majorities). Γκέι γυναίκες o Γκέι άντρες o Αμφιφυλόφιλοι o Τρανσέξουαλς o				Σωστό	Λάθος
46.	Οι σεξουαλικές μειονότητες αντιμετωπίζουν μεγαλύτερο κίνδυνο για χρόνιες παθήσεις από ό,τι οι ετεροφυλόφιλοι & οι σεξουαλικές πλειοψηφίες (gender majorities). Γκέι γυναίκες o Γκέι άντρες o Αμφιφυλόφιλοι o Τρανσέξουαλς o				Σωστό	Λάθος
47.	Οι σεξουαλικές μειονότητες αντιμετωπίζουν μεγαλύτερο κίνδυνο για σεξουαλικώς μεταδιδόμενα νοσήματα από ό,τι οι ετεροφυλόφιλοι & οι σεξουαλικές πλειοψηφίες (gender majorities). Γκέι γυναίκες o Γκέι άντρες o Αμφιφυλόφιλοι o Τρανσέξουαλς o				Σωστό	Λάθος
48.	Οι σεξουαλικές μειονότητες βιώνουν υψηλότερα ποσοστά σωματικού και σεξουαλικού τραύματος από ό,τι οι ετεροφυλόφιλοι & οι σεξουαλικές πλειοψηφίες (gender majorities). Γκέι γυναίκες o Γκέι άντρες o Αμφιφυλόφιλοι o Τρανσέξουαλς o				Σωστό	Λάθος
Παρακαλώ, διαβάστε κάθε δήλωση και σημειώστε τον βαθμό συμφωνίας ή διαφωνίας σας στην παρακάτω κλίμακα. Επιλέξτε μια απάντηση ανά σειρά.						
49.	Κατά την άσκηση των καθηκόντων τους με ΛΟΑΤKI άτομα, οι επαγγελματίες υγείας θα πρέπει να υποστηρίζουν την ποικιλόμορφη σύνθεση των οικογενειών τους	Διαφωνώ έντονα	Διαφωνώ	Ουδέτερος/-η ούτε διαφωνώ/ούτε συμφωνώ	Συμφωνώ	Συμφωνώ έντονα
50.	Οι επαγγελματίες υγείας θα πρέπει να εκφράζουν τον σεβασμό τους για τον τρόπο ζωής των ΛΟΑΤKI ατόμων-ασθενών που καλούνται να φροντίσουν	Διαφωνώ έντονα	Διαφωνώ	Ουδέτερος/-η ούτε διαφωνώ/ούτε συμφωνώ	Συμφωνώ	Συμφωνώ έντονα
51.	Οι επαγγελματίες υγείας θα πρέπει να καταβάλλουν προσπάθεια να μάθουν για τη διαφορετικότητα (diversity) στη ΛΟΑΤKI κοινότητα	Διαφωνώ έντονα	Διαφωνώ	Ουδέτερος/-η ούτε διαφωνώ/ούτε συμφωνώ	Συμφωνώ	Συμφωνώ έντονα
52.	Οι επαγγελματίες υγείας θα πρέπει να γνωρίζουν τους πόρους/ φορείς υποστήριξης για τα ΛΟΑΤKI άτομα	Διαφωνώ έντονα	Διαφωνώ	Ουδέτερος/-η ούτε διαφωνώ/ούτε συμφωνώ	Συμφωνώ	Συμφωνώ έντονα
53.	Οι επαγγελματίες υγείας θα πρέπει να εκπαιδεύονται σχετικά με τον τρόπο ζωής των ΛΟΑΤKI ατόμων	Διαφωνώ έντονα	Διαφωνώ	Ουδέτερος/-η ούτε διαφωνώ/ούτε συμφωνώ	Συμφωνώ	Συμφωνώ έντονα
54.	Οι επαγγελματίες υγείας θα πρέπει να αμφισβητούν την παραπληροφόρη ση σχετικά με τα ΛΟΑΤKI άτομα	Διαφωνώ έντονα	Διαφωνώ	Ουδέτερος/-η ούτε διαφωνώ/ούτε συμφωνώ	Συμφωνώ	Συμφωνώ έντονα
55.	Οι επαγγελματίες υγείας θα πρέπει να χρησιμοποιούν τις ευκαιρίες επαγγελματικής ανάπτυξης για να βελτιώσουν την παρεχόμενη υγειονομική φροντίδα στα ΛΟΑΤKI άτομα	Διαφωνώ έντονα	Διαφωνώ	Ουδέτερος/-η ούτε διαφωνώ/ούτε συμφωνώ	Συμφωνώ	Συμφωνώ έντονα
56.	Οι επαγγελματίες υγείας θα πρέπει να είναι γνώστες των ζητημάτων/ αναγκών υγείας που αφορούν αποκλειστικά τα ΛΟΑΤKI άτομα	Διαφωνώ έντονα	Διαφωνώ	Ουδέτερος/-η ούτε διαφωνώ/ούτε συμφωνώ	Συμφωνώ	Συμφωνώ έντονα
57.	Οι επαγγελματίες υγείας θα πρέπει να αποκτήσουν τις απαραίτητες γνώσεις για την αποτελεσματική φροντίδα των ΛΟΑΤKI ατόμων/ ασθενών	Διαφωνώ έντονα	Διαφωνώ	Ουδέτερος/-η ούτε διαφωνώ/ούτε συμφωνώ	Συμφωνώ	Συμφωνώ έντονα
58.	Οι επαγγελματίες υγείας θα πρέπει να εργαστούν για να αναπτύξουν τις δεξιότητες που είναι απαραίτητες για την αποτελεσματική φροντίδα των ΛΟΑΤKI ατόμων/ ασθενών	Διαφωνώ έντονα	Διαφωνώ	Ουδέτερος/-η ούτε διαφωνώ/ούτε συμφωνώ	Συμφωνώ	Συμφωνώ έντονα
59.	Οι επαγγελματίες υγείας θα πρέπει να εργαστούν για να αναπτύξουν συμπεριφορές που είναι απαραίτητες για την αποτελεσματική πρακτική/ φροντίδα των ΛΟΑΤKI ατόμων/ ασθενών	Διαφωνώ έντονα	Διαφωνώ	Ουδέτερος/-η ούτε διαφωνώ/ούτε συμφωνώ	Συμφωνώ	Συμφωνώ έντονα
60.	Η πολιτική της «μη διάκρισης των ασθενών» είναι απαραίτητη για την εξασφάλιση ασφαλούς και ποιοτικής θεραπείας όλων των ασθενών	Διαφωνώ έντονα	Διαφωνώ	Ουδέτερος/-η ούτε διαφωνώ/ούτε συμφωνώ	Συμφωνώ	Συμφωνώ έντονα
61.	Η πολιτική της «μη διάκρισης των ασθενών» θα πρέπει να κοινοποιείται στους ασθενείς και τους εργαζόμενους	Διαφωνώ έντονα	Διαφωνώ	Ουδέτερος/-η ούτε διαφωνώ/ούτε συμφωνώ	Συμφωνώ	Συμφωνώ έντονα
62.	Η πολιτική επισκεπτηρίου θα πρέπει να παρέχει ρητά ισότιμες δυνατότητες επίσκεψης στους ΛΟΑΤKI ασθενείς και τους επισκέπτες τους	Διαφωνώ έντονα	Διαφωνώ	Ουδέτερος/-η ούτε διαφωνώ/ούτε συμφωνώ	Συμφωνώ	Συμφωνώ έντονα
63.	Η εκπαίδευση του προσωπικού και των επαγγελματιών υγείας σε θέματα φροντίδας με επίκεντρο τους ΛΟΑΤΚΙ ασθενείς, βελτιώνει την ποιότητα της φροντίδας που τους παρέχεται	Διαφωνώ έντονα	Διαφωνώ	Ουδέτερος/-η ούτε διαφωνώ/ούτε συμφωνώ	Συμφωνώ	Συμφωνώ έντονα
Παρακαλώ, σημειώστε εάν οι παρακάτω δηλώσεις είναι σωστές ή λάθος, αναφορικά με τη δομή υγείας στην οποία εργάζεστε. Επιλέξτε μια απάντηση ανά σειρά						
64.	Στην πολιτική των δικαιωμάτων των ασθενών περιλαμβάνεται ο όρος «σεξουαλικός προσανατολισμός»				Σωστό	Λάθος
65.	Στην πολιτική των δικαιωμάτων των ασθενών περιλαμβάνεται ο όρος «ταυτότητα φύλου»				Σωστό	Λάθος
66.	Η πολιτική της «μη διάκρισης των ασθενών» κοινοποιείται στους ασθενείς				Σωστό	Λάθος
67.	Η πολιτική της «μη διάκρισης των ασθενών» κοινοποιείται στους εργαζομένους				Σωστό	Λάθος
68.	Η πολιτική επισκεπτηρίου χορηγεί ρητά ισότιμες δυνατότητες επίσκεψης σε ασθενείς ΛΟΑΤΚΙ και τους επισκέπτες τους				Σωστό	Λάθος
69.	Η πολιτική επισκεπτηρίου κοινοποιείται στους ασθενείς και στους επισκέπτες				Σωστό	Λάθος
70.	Στην πολιτική της «μη διάκρισης των εργαζομένων» περιλαμβάνεται ο όρος «σεξουαλικός προσανατολισμός»				Σωστό	Λάθος
71.	Στην πολιτική της «μη διάκρισης των εργαζομένων» περιλαμβάνεται ο όρος «ταυτότητα φύλου»				Σωστό	Λάθος
72.	Το προσωπικό της δομής υγείας εκπαιδεύεται στην ασθενο-κεντρική φροντίδα (patient-centered care) των ΛΟΑΤΚΙ ασθενών				Σωστό	Λάθος
Απ΄ όσο γνωρίζετε, στη δομή υγείας που εργάζεστε:						
73.	Τι ποσοστό των ασθενών που εξυπηρετεί η δομή υγείας σας, εκτιμάτε ότι είναι ομοφυλόφυλοι ή αμφιφυλόφιλοι; ____________%					
74.	Τι ποσοστό των ασθενών που εξυπηρετεί η δομή υγείας σας, εκτιμάτε ότι είναι τρανσέξουαλ (transgender); ____________%					
Επιπρόσθετες ερωτήσεις						
1.	Πόσων ετών είστε?________					
2.	Πότε ολοκληρώσατε τις προπτυχιακές σας σπουδές στη Μαιευτική? ______ (έτος)					
3.	Παρακαλώ σημειώστε τυχόν επιπλέον σπουδές: α. Κάτοχος Μεταπτυχιακού τίτλου σπουδών β. Κάτοχος Διδακτορικού τίτλου σπουδών γ. Κάτοχος άλλου πτυχίου ΑΕΙ					
4.	Εργάζεστε σε: α. Δημόσιο Νοσηλευτικό ίδρυμα β. Ιδιωτικό Νοσηλευτικό Ίδρυμα γ. Ελεύθερος Επαγγελματίας δ. Ιδιωτικός υπάλληλος σε γραφείο ε. Στην εκπαίδευση στ. Αλλού: _____________________					
5.	Εάν εργάζεστε σε Νοσοκομείο, σε ποιο Τμήμα/ Πτέρυγα εργάζεστε; α. Αίθουσα Τοκετών/ Παραλαβή β. Τακτικά Εξωτερικά Μαιευτικά/ Γυναικολογικά Ιατρεία γ. Μονάδα Νεογνών δ. Τμήμα Λεχωΐδων ε. Τμήμα Υποβοηθούμενης Αναπαραγωγής στ. Τμήμα Υπερήχων ζ. Χειρουργείο η. Τμήμα Κυήσεων Υψηλού Κινδύνου θ. Διοικητική Θέση ι. Κέντρο Υγείας ια. Αλλού……………………………………….					
6.	Πόσο τακτικά θεωρείτε ότι έχετε έρθει σε επαφή με ΛΟΑΤΚΙ+ άτομα/ ασθενείς στον επαγγελματικό σας χώρο στη διάρκεια των τελευταίων 12 μηνών; α. Ποτέ β. 1 φορά/ 6μήνες γ. 1 φορά/ μήνα δ. 1 φορά/ εβδομάδα ε. Δε γνωρίζω					
7.	Κατά τη διάρκεια λήψης ατομικού ιστορικού σε ενήλικο άτομο ρωτάτε το φύλο και τον σεξουαλικό προσανατολισμό του; α. Ναι β. Όχι γ. Ανάλογα με την εικόνα του					

**Table 9:** The English translation of the Greek version of the PKSGMH instrument used for the study PKSGMH: Perception and Knowledge of Sexual and Gender Minority Health

Evaluation tool for perceptions and knowledge on the health of sexual and gender minorities (English translation of the Greek version)						
1.	How many years have you been practiced as a midwife/health care provider? ______ years					
2.	Gender					
	a. Male b. Female c. Transgender d. Prefer not to answer					
3.	Sexual orientation					
	a. Heterosexual b. Homosexual c. Bisexual d. Prefer not to answer					
4.	Marital status					
	a. Married b. In a long-term relationship c. Single d. Divorced e. Widowed					
5.	Do you have acquaintances, peers, colleagues, friends, or family members who belong to the LGBTQ+ community?					
	a. Yes b. No					
6.	Do you provide primary care to individuals/patients who belong to a sexual minority or the LGBTQ+ community? Select all that apply:					
	a. Lesbian b. Gay c. Bisexual d. Transgender					
7.	Have you received focused training on the care of LGBTQ+ individuals/patients?					
	a. Yes b. No					
8.	If yes, where and when did you receive training on the care of LGBTQ+ individuals/patients?					
	Where: __________________________________________________________					
	When: __/__/__ (dd/mm/yy)					
Next are some questions about your opinions on Homosexual, Bisexual, and Transgender Individuals. Please read each statement and indicate your level of agreement or disagreement on the scale below. All responses are kept confidential.						
	Attitudes toward gay men. Mark one answer per row					
9.	Sex between two men is simply wrong	Strongly disagree	Disagree	Neither agree nor disagree	Agree	Strongly agree
10.	I find gay men disgusting	Strongly disagree	Disagree	Neither agree nor disagree	Agree	Strongly agree
11.	Male homosexuality is a natural expression of male sexuality	Strongly disagree	Disagree	Neither agree nor disagree	Agree	Strongly agree
	Attitudes toward lesbians. Mark one answer per row					
12.	Sex between two women is simply wrong	Strongly disagree	Disagree	Neither agree nor disagree	Agree	Strongly agree
13.	I find lesbian women disgusting	Strongly disagree	Disagree	Neither agree nor disagree	Agree	Strongly agree
14.	Female homosexuality is a natural expression of female sexuality	Strongly disagree	Disagree	Neither agree nor disagree	Agree	Strongly agree
	Attitudes toward bisexuals. Mark one answer per row					
15.	Having sex with both men and women is simply wrong	Strongly disagree	Disagree	Neither agree nor disagree	Agree	Strongly agree
16.	I find bisexual individuals disgusting	Strongly disagree	Disagree	Neither agree nor disagree	Agree	Strongly agree
17.	Bisexuality is a natural expression of sexuality in both men and women	Strongly disagree	Disagree	Neither agree nor disagree	Agree	Strongly agree
	Attitudes toward transgender individuals. Mark one answer per row.					
18.	It is simply wrong for a person’s gender identity (male or female) not to align with their biological/ assigned sex at birth (male or female).	Strongly disagree	Disagree	Neither agree nor disagree	Agree	Strongly agree
19.	I find transgender individuals disgusting	Strongly disagree	Disagree	Neither agree nor disagree	Agree	Strongly agree
20.	Being transgender is a natural expression of gender identity in men and women	Strongly disagree	Disagree	Neither agree nor disagree	Agree	Strongly agree
The following questions concern your feelings about providing healthcare to LGBTQ+ Patients. Please read each statement and indicate your level of agreement or disagreement on the scale below. All responses are kept confidential.						
21.	I would prefer not to provide care to LGBTQ+ patients	Strongly disagree	Disagree	Neither agree nor disagree	Agree	Strongly agree
22.	I would refuse care for an LGBTQ+ patient if I knew they identified as LGBTQ+	Strongly disagree	Disagree	Neither agree nor disagree	Agree	Strongly agree
23.	I feel competent in providing care to LGBTQ+ patients.	Strongly disagree	Disagree	Neither agree nor disagree	Agree	Strongly agree
24.	LGBTQ+ patients do not have specific healthcare needs	Strongly disagree	Disagree	Neither agree nor disagree	Agree	Strongly agree
25.	I feel that I could communicate with an LGBTQ+ patient in a sensitive and appropriate way	Strongly disagree	Disagree	Neither agree nor disagree	Agree	Strongly agree
26.	I believe that my undergraduate education adequately covers the healthcare needs of the LGBTQ+ population	Strongly disagree	Disagree	Neither agree nor disagree	Agree	Strongly agree
Please mark whether you believe the following statements are true or false						
27.	The terms “sex” and “gender” have the same meaning				True	False
28.	Most homosexual individuals want to be members of the opposite sex				True	False
29.	Gay men always behave and dress femininely				True	False
30.	Gay men are more likely to be victims of violent crimes compared to the general population				True	False
31.	Bisexual individuals will eventually “come out” as homosexuals				True	False
32.	Bisexual behavior is often just a “cry for attention”				True	False
33.	To be considered transgender, a person must have undergone a sexual reassignment surgery				True	False
34.	Transgender women (male-to-female) are always attracted to individuals with male genitalia				True	False
35.	A transgender person should be addressed using their preferred gender pronouns rather than their biological sex				True	False
36.	Lesbians always dress and behave masculinely				True	False
37.	LGBTQ+ patients seek medical care later than heterosexuals due to fear of discrimination				True	False
38.	Most healthcare professionals automatically assume their patient is heterosexual unless explicitly stated otherwise				True	False
39.	LGBTQ+ individuals may exhibit signs of depression due to a lack of social acceptance				True	False
Please indicate whether the statements are True or False. If they are True, please mark for which subgroups the statement is True (you can mark more than one subgroup).						
40.	Sexual/ Gender minorities experience a higher prevalence of depression and anxiety than heterosexuals. Lesbian o Gay o Bisexual o Transgender o				True	False
41.	Sexual/ Gender minorities have higher rates of alcohol use and alcohol-related problems than heterosexuals and gender-majorities. Lesbian o Gay o Bisexual o Transgender o				True	False
42.	Sexual/ Gender minorities are twice as likely to use tobacco compared to heterosexuals and gender-majorities. Lesbian o Gay o Bisexual o Transgender o				True	False
43.	Sexual/ Gender minorities report higher substance use than heterosexuals and gender-majorities. Lesbian o Gay o Bisexual o Transgender o				True	False
44.	Sexual minorities have less access to healthcare services than heterosexuals and gender-majorities. Lesbian o Gay o Bisexual o Transgender o				True	False
45.	Sexual minorities have higher rates of obesity/overweight compared to heterosexuals and gender-majorities. Lesbian o Gay o Bisexual o Transgender o				True	False
46.	Sexual minorities experience greater risk for chronic diseases than heterosexuals and gender-majorities. Lesbian o Gay o Bisexual o Transgender o				True	False
47.	Sexual minorities experience greater risk for sexually transmitted infections than heterosexuals and gender-majorities. Lesbian o Gay o Bisexual o Transgender o				True	False
48.	Sexual minorities experience higher rates of physical and sexual trauma than heterosexuals and gender-majorities. Lesbian o Gay o Bisexual o Transgender o				True	False
Please read each statement and indicate your level of agreement or disagreement on the scale below. Mark one answer per row.						
49.	In their practice with LGBTQ+ clients, healthcare professionals should support the diverse composition of their families	Strongly disagree	Disagree	Neither agree nor disagree	Agree	Strongly agree
50.	Healthcare professionals should verbalize respect for the lifestyles of LGBTQ+ individuals/patients	Strongly disagree	Disagree	Neither agree nor disagree	Agree	Strongly agree
51.	Healthcare professionals should make an effort to learn about diversity within the LGBTQ+ community	Strongly disagree	Disagree	Neither agree nor disagree	Agree	Strongly agree
52.	Healthcare professionals should be aware of LGBTQ+ support organizations/resources	Strongly disagree	Disagree	Neither agree nor disagree	Agree	Strongly agree
53.	Healthcare professionals should educate themselves about LGBTQ+ lifestyles	Strongly disagree	Disagree	Neither agree nor Disagree	Agree	Strongly agree
54.	Healthcare professionals should challenge misinformation about LGBTQ+ individuals/ patients	Strongly disagree	Disagree	Neither agree nor disagree	Agree	Strongly agree
55.	Healthcare professionals should use professional development opportunities to improve their practice for LGBTQ+ individuals	Strongly disagree	Disagree	Neither agree nor disagree	Agree	Strongly agree
56.	Healthcare professionals should be knowledgeable about health issues unique to LGBTQ+ individuals	Strongly disagree	Disagree	Neither agree nor disagree	Agree	Strongly agree
57.	Healthcare professionals should acquire the necessary knowledge for effective LGBTQ+ patient care	Strongly disagree	Disagree	Neither agree nor disagree	Agree	Strongly agree
58.	Healthcare professionals should work to develop the skills necessary for effective LGBTQ+ patient care	Strongly disagree	Disagree	Neither agree nor disagree	Agree	Strongly agree
59.	Healthcare professionals should work to develop attitudes necessary for effective LGBTQ+ patient care	Strongly disagree	Disagree	Neither agree nor disagree	Agree	Strongly agree
60.	A "non-discrimination policy" is essential to ensure safe and quality treatment for all patients	Strongly disagree	Disagree	Neither agree nor disagree	Agree	Strongly agree
61.	Patient "non-discrimination policy" should be communicated to patients and staff	Strongly disagree	Disagree	Neither agree nor disagree	Agree	Strongly agree
62.	The hospital visitation policy should explicitly allow equal visitation rights for LGBTQ+ patients and their visitors	Strongly disagree	Disagree	Neither agree nor disagree	Agree	Strongly agree
63.	Training healthcare professionals in LGBTQ+-centered care improves the quality of care provided to LGBTQ+ patients	Strongly disagree	Disagree	Neither agree nor disagree	Agree	Strongly agree
Workplace policies and practices indicate whether the following statements are True or False, in relation to the health facility where you work. Mark one answer per row						
64.	The patient's rights policy includes the term "sexual orientation"				True	False
65.	The patient's rights policy includes the term "gender identity"				True	False
66.	The non-discrimination policy is communicated to patients				True	False
67.	The non-discrimination policy is communicated to employees				True	False
68.	The hospital visitation policy explicitly allows equal visitation rights for LGBTQ+ patients and visitors				True	False
69.	The visitation policy is communicated to patients and visitors				True	False
70.	The employee non-discrimination policy includes the term "sexual orientation"				True	False
71.	The employee non-discrimination policy includes the term "gender identity"				True	False
72.	Healthcare staff receive training in patient-centered care for LGBTQ+ patients				True	False
As far as you know, in the healthcare facility where you work:						
73.	What percentage of the patients served by your healthcare facility do you estimate are homosexual or bisexual? ____________%					
74.	What percentage of the patients served by your healthcare facility do you estimate are transgender? ____________%					
Additional questions						
1.	How old are you? _______					
2.	When did you complete your undergraduate studies in Midwifery? ______ (year)					
3.	Please indicate any additional studies: a. Holder of a Master's degree b. Holder of a Ph.D. c. Holder of another university degree					
4.	Where do you work? a. Public healthcare institution b. Private healthcare institution c. Self-employed d. Private sector employee (office-based) e. In education f. Other________________					
5.	If you work in a hospital, in which department/ward do you work? a. Delivery Room/Admission b. Outpatient Obstetrics/Gynecology Clinics c. Neonatal Unit d. Postnatal Ward e. Assisted Reproduction Unit f. Ultrasound Department g. Surgery h. High-Risk Pregnancy Unit i. Administrative Position j. Health Center k. Other: _______________________________					
6.	How often have you encountered LGBTQ+ individuals/patients in your professional setting in the past 12 months? a. Never b. Once every 6 months c. Once a month d. Once a week e. I don't know					
7.	When taking an individual medical history for an adult patient, do you ask about their gender and sexual orientation? a. Yes b. No c. Depending on their appearance					
